# Association of *HTR3B* gene polymorphisms with depression and its executive dysfunction: a case–control study

**DOI:** 10.1186/s12888-023-04625-y

**Published:** 2023-02-27

**Authors:** Lina Wang, Miao Wang, Chaoben Zhao, Jia Jian, Dongdong Qiao

**Affiliations:** 1 Department of Psychology, Shandong Normal University, Jinan, 250358 Shandong China; 2 Department of Psychiatry, Shandong Mental Health Center, Shandong University, Jinan, 250014 Shandong China; 3 School of Mental Health, Jining Medical University, Jining, 272000 Shandong China

**Keywords:** *HTR3B*, Polymorphism, Depression, Executive dysfunction

## Abstract

**Background:**

Previous studies have shown that depression was associated with *HTR3B* gene. The aim of this study was to investigate the relationship between polymorphisms of the *HTR3B* gene and depression and its executive dysfunction in Chinese Han population.

**Methods:**

A total of 229 patients with depressive disorder and 202 healthy controls were enrolled. Six Single nucleotide polymorphism sites (SNPs) including rs10789970, rs4938056, rs12421126, rs1176744, rs2276305 and rs12795805 were genotyped by Snapshot. Clinical features were collected using a general demographic questionnaire. The 24-item Hamilton Depression Scale (HAMD) was used to assess the symptoms’ severity of the patients. The patients' executive function was assessed using a series of cognitive tests including Maze Test, Symbolic Coding Test, Spatial Span Inverse Order Test, Linking Test, and Emotional Management Test.

**Results:**

The genotypic and allelic distributions of rs1176744 in *HTR3B* gene were significantly different (χ^2^ = 11.129, *P* = 0.004, χ^2^ = 9.288, *P* = 0.002, respectively) between patients and controls. The A allele was positively correlated with depression. The proportion of A carriers was significantly higher and that of C carriers was lower in patients than those in controls. Patients had significantly lower scores of Spatial Span Inverse Order Test in carriers of A allele at locus rs1176744 and higher scores in carriers of C alleles at locus rs1176744 and rs12795805.

**Conclusions:**

The polymorphisms of *HTR3B* gene may be associated with depression in Chinese Han population. The A allele of rs1176744 may increase the risk of developing depression and executive dysfunction while C alleles of rs1176744 and rs12795805 may be the protective factors for executive dysfunction in patients with depression.

## Introduction

Major Depressive Disorder (MDD) is the leading cause of disability [1] according to the World Health Organization (WHO), which affects roughly 350 million people worldwide [2], and is the third-largest contributor to the global burden of disease [3]. Numerous studies have demonstrated that cognitive dysfunction in patients with major depression is a strong predictor of occupational and social dysfunction [4]. The Diagnostic and Statistical Manual of Mental Disorders, Fifth Edition(DSM5) states that specific criteria for depression include cognitive function issues such as impaired attention, memory, and executive functioning [5]. Patients with MDD experience permanent and recurrent cognitive impairment leading to significantly lower quality of life, and these deficiencies may continue even after a major depressive episode has subsided [6]. These deficits include deficits in the domains of processing speed, visual selective attention, working memory, verbal learning, and executive function [7]. Studies have reported that approximately 20–30% of depressed individuals have severe executive function deficits [8], which have been shown to be the strongest independent predictor of functioning in patients with MDD in remission [9]. And a brief cognitive screen at the start of treatment, focusing on executive functioning, was considered to be of useful value in the prognosis of depression [10].

MDD is thought to be a complex polygenetic disorder in which both genetic and environmental factors are involved in its onset and development [11, 12]. The focus of current genetic research is mostly on functional genes such as genes from dopamine system and the serotonergic gene variants (i.e., *HTR1A-7 and SLC6A4*) [13, 14]. 5-HT receptors (HTR) are known to be involved in the etiology of depression. Currently, it is known that a total of 18 genes are responsible for 14 different mammalian 5-HT receptor subtypes, which are further subdivided into 7 families, and the 5-HT3 receptor, which is a distinct ion channel [15], is expressed in both the central nervous system (CNS) and the peripheral nervous system (PNS) and has the ability to control rapid depolarization of either peripheral or central neurons [16]. Presynaptic 5-HT3 receptor activation is followed by rapid depolarization, which causes a rapid rise in cytosolic Ca^2+^ concentration. Postsynaptic activation results in depolarization by Na^+^ and K^+^ influx, which regulates CNS and PNS function [17]. A previous study showed that the antagonist of the 5-HT3 receptor could reverse depressive-like behavior [18]. Ondansetron, a serotonin 5-HT3 receptor antagonist, has been shown to boost the expression of antioxidant components like glutathione, increase the levels of 5-HT, and reduce depression in diabetic rats by inhibiting the 5-HT3 receptor [19].

Five subunits of the 5-HT3 receptor make up the ligand-gated ion channel which are encoded by the serotonin receptor genes *HTR3A*, *HTR3B*, *HTR3C*, *HTR3D*, and *HTR3E*, respectively [20]. 5-HT3B [21] is one of the most well investigated and well-characterized subunits which may have a role in tissue-specific functional alterations in 5-HT3-mediated signaling and/or regulation [20]. A clinical study found that the polymorphism of rs1176744 in *HTR3B* gene was related to major depression in women [22]. Another study in 2019 discovered a link between rs1176744 polymorphism and major depression in the Russian population [23]. Meanwhile, *HTR3B* rs1176744 polymorphism and rs3831455 deletion have been linked to bipolar disorder in various studies [22, 24, 25]. The 5-HTR3 antagonist Ondansetron has been shown to be able to counteract scopolamine-induced learning deficits [26] and improve maze performance [27]. Itasetron, another 5-HTR3 antagonist, has been found to have memory-enhancing effects [28]. So far, there is no study exploring the relationship between the polymorphisms of *HTR3B* gene and cognitive dysfunction in patients with depression.

Therefore, we conducted a case–control study to investigate the association between *HTR3B* gene polymorphisms and depression and its executive function in Chinese Han patients with major depressive disorder.

## Methods

### Participants

In this study, patients who visited outpatient clinics or were admitted to hospital in Shandong Mental Health Center from January 2012 to March 2014 were enrolled. The inclusion criteria included: patients met the Diagnostic and Statistical Manual of Mental Disorders—Fourth Edition (DSM-IV) diagnosis of major depressive disorder; male or female; aged between 30 and 60 years old; biological parents were Chinese Han population; 24-item Hamilton Depression Scale (HAMD) score ≥ 20. Depressive episodes caused by organic brain disorders, psychoactive substance use, severe physical illnesses, and other psychiatric disorders were all excluded. Other exclusion criteria included women who were pregnant or nursing, those with a history of mild mania, manic or mixed episodes, or those with psychotic symptoms. Healthy adults who were aged between 30 and 60 years old were included in the study as the control group. They also met the following criteria: biological parents were Chinese Han population; had no neurological illness or personality disorder which match the DSM-IV axis I and axis II diagnoses; had no family history of mental illness. Each participant gave their informed consent in writing and took part in the study voluntarily. Ethical approval was granted by the ethics committee of Shandong Mental Health Center.

### Assessments

General sociodemographic data and personal history of all the participants were collected using a self-made questionnaire which included age, gender and years of education. The 24-item Hamilton Depression Scale (HAMD) was used to evaluate the symptoms’ severity of the patients.

The patients' executive function was assessed using the MATRICS Consensus Cognitive Battery (MCCB), a computerized testing package, which has been validated in China and has a good internal consistency and acceptable reliability for patients with mood disorder [29]. The specific tests used are as follows.

- Maze Test (MT): The purpose of the test is to assess reasoning and problem-solving skills, including anticipation, planning, and impulse control. The test is administered by drawing a pathway from the entrance to the exit of the maze. The lines should be drawn in accordance with the requirements and should not intersect with the original lines in the maze, nor should the lines be drawn at the corners. If the subject does not get out of the maze within the time limit, a score of 0 will be given. If the subject comes out of the maze within the specified time, the score will be given according to the time he/she actually completed the maze. All the scores of the maze will be added up to the raw score.
- Symbolic Coding Test (SCT): Using the standard template provided as a reference, the participant selects the numbers that match the different symbols and fills in the blanks, requiring the participant to complete them within 90 s. The number of errors is subtracted from the total number of answers to give the final score.
- Spatial Span Inverse Order Test (SSIOT): The main test is spatial location memory breadth. Ten blue squares are displayed on the screen and the participant is asked to click on them repeatedly in the opposite order. A score of 1 is given to those who click in exactly the right order. Those who click on more or fewer squares or in the wrong order will receive 0 points.
- Linking Test (LT): The test is designed to assess visual scanning and motion trajectories that reflect the participant's speed of information processing. The participant is asked to connect the numbers on the answer key in order from smallest to largest without interruptions during the test. The participant's completion time is used as the scoring criterion. If the participant exceeds the time limit, the test time is recorded as 300 s and those who do not finish within the time limit are scored as errors.
- Emotional Management Test (EMT): This test is designed to assess the ability to use emotions to accomplish tasks and solve emotional problems, and the ability to integrate their emotions into problem-solving. The total score is used as the raw score.

### SNPs selection

SNPs located at the 3' end, 5' end, or exon region were selected based on the confirmed *HTR3B* gene sequence information from the National Center for Biotechnology Information (NCBI) database. We selected SNPs which had a minimum allele frequency (MAF) greater than 10% in the Chinese Han population. Six loci of *HTR3B* gene (rs10789970, rs4938056, rs12421126, rs1176744, rs2276305 and rs12795805) were chosen, the MAF of which were 42.5%, 43.7%, 21.7%, 18.6%, 23.2% and 15.9%, respectively.

### DNA extraction and SNPs genotyping

First, 5 ml of venous blood of all participants were drawn and stored in an anticoagulant tube (with EDTA anticoagulant). The collected samples were then centrifuged at the speed of 3000 RPM for 10 min. After centrifugation, leukocytes were extracted and placed in EP tubes in -70℃ refrigerator for use.

The whole genome DNA was extracted from peripheral blood leukocytes using the DNA extraction kit from Hangzhou Jiuna Biotechnology Co., Ltd. A total of 1500 μl of red blood cell lysate was added into the above blood samples after thawing and centrifuged at 12,000 rpm for 20 s. After that, 500 μl of cell nucleus lysate and 200 μl of protein precipitate was added and centrifuged at 13,000 rpm for 5 min, then 650 μl of propanol was added to obtain a filamentous or cotton wool-like white DNA precipitate. Then, added 1 ml 70% ethanol to the DNA precipitate and centrifuged at 12,000 rpm for 1 min, and added 0.5 ml 170% ethanol to centrifuge at 12,000 μl for 1 min. Finally, the precipitate was wiped dry and 50 μl sterile water was added to dissolve the DNA precipitate, and the extracted DNA was then put into the -70 ℃ refrigerator again for use.

The primers were designed using the online primer design tool and synthesized by Beijing Genomics institution (BGI) (Table 1). The PCR was carried out in a 5-μl volume containing 1 μl genomic DNA, 0.5 μl of 10 × PCR buffer, 1 μl of Primer, 0.1 μl of dNTPs (2.5 mM each), 1.8 μl of HPLC and 0.2 μl of Ex Taq [5U/μl], 0.4 μl of 25 mM MgCI_2_. The reaction conditions of PCR were as follows: after an initial step of 15 min at 94 °C, denaturation at 94 °C for 20 s, annealing at 56 °C for 30 s, extended at 72 °C for 60 s; 45 cycles in total. Then the amplified products were digested and purified. The SAP (Shrimp Alkaline Phosphatase) reaction was carried out in a volume containing 1.53 μl of deionized water, 0.17 μl of SAP buffer and 0.3 μl of SAP enzyme 2μL. The reaction conditions for SAP were as follows: after an initial step of 40 min at 37 °C, the SAP reaction was completed at a temperature of 4 °C after a continuous period of 5 min at a temperature of 85 °C. Then, the Snapshot extension reactions were performed with the Snapshot Multiplex PCR Kit, and the 9 μl volume containing 7 μl of PCR product, 0.041 μl iplex enzyme, 0.94 μl of iplex extension primer, 0.2 μl of iplex stop primer, 0.2 μl of iplex buffer and 0.619 μl of deionized water. After an initial step of 30 s at 94 °C, denaturation at 94 °C for 5 s, annealing at 52 °C for 5 s, extended at 80 °C for 5 s and then followed by 40 cycles. Finally, data analyses and genotyping were performed using Mass ARRAY software after desalination.

**Table 1:** Primer sequences

SNP	Upstream primers	Downstream primers	PCR length
rs10789970	ACGTTGGATGTCTATTCAGGAG GAAACACC	ACGTTGGATGGGCACTTTTGAAG ATGCCTG	98
rs4938056	ACGTTGGATGAGGCGACAAGAT CAAGACTC	ACGTTGGATGGAGAGCTTCAGTT TCTCCAC	112
rsl2421126	ACGTTGGATGGGAAATGCAAAC AACCTAGA	ACGTTGGATGGTAGCATTATGGA AGTCTTG	119
rsl176744	ACGTTGGATGTGGTCCCAGATG AGTTCAC	ACGTTGGATGCTCTGTGACAACA AGTTCTC	114
rs2276305	ACGTTGGATGACCCTCAGCCTG AGATCCA	ACGTTGGATGAGGGTTTCTCCTC CACTATC	109
rsl2795805	ACGTTGGATGCAGCACAGGTTA TTATTCAC	ACGTTGGATGTCAGAAGGTGAG GGATATGG	116

### Statistical analysis

Statistical description and analyses were carried out using SPSS 21.0 software.

The goodness of fit Chi-square test was used to compare the allelic genotypes of six SNPs and test whether they conformed to Hardy Weinberg equilibrium (HWE). Chi-square test and Independent-samples t-test were used to compare the differences of demographic data between patients and healthy controls, where independent samples t-tests were also used to compare the cognitive function test scores of carriers of different alleles at different genetic loci. One way ANOVA and Pearson correlation were used to analyze the relationship between general information and cognitive function. Covariance analysis was used to analyze the association between different genotypes of gene polymorphisms and cognitive dysfunction. The difference was statistically significant when *Ρ* < 0.05. Bonferroni correction was used to calculate the threshold P value for significance tests. Chain imbalance and haplotype analyses were carried out using the Haploview software (version 4.2).

## Results

### General demographic characteristics of the participants

A total of 229 patients with depressive disorder were enrolled in this study, in which 106 patients were male and 123 were female. The control group consisted of 202 participants, including 90 males and 112 females. There was no statistical difference between the two groups in terms of age, sex, or years of education (*P* > 0.05) (Table 2).

**Table 2:** Comparison of general demographic information between patients and controls

	patients	Healthy Controls	χ^2^/t value	*P*-value
Gender			0.130	0.718
Male	106	90		
Female	123	112		
Age (years)	42.47 ± 8.15	42.55 ± 8.32	-0.104	0.917
Education (years)	12.39 ± 4.39	12.64 ± 5.18	-0.543	0.587

### Association of the *HTR3B* gene polymorphisms with depressive disorder

#### Hardy–Weinberg Law of Equilibrium

The *HTR3B* gene's six polymorphic loci were tested using the Hardy–Weinberg law of Equilibrium in both the case and control groups. The genotype distribution of five loci (rs10789970, rs4938056, rs12421126, rs1176744 and rs12795805) except for rs2276305 (*P* < 0.05) were consistent with the Hardy–Weinberg Equilibrium. The genetic data of rs2276305 was excluded from our following study.

#### Genotypic distributions of *HTR3B* gene in patients and controls

The genotypic distribution of rs1176744 in *HTR3B* gene was statistically different (χ^2^ = 11.129, *P* = 0.004) between patients and controls. The difference remained significant after Bonferroni correction (*P* < 0.01correcting for 5 tests). The frequencies of CC and AC genotypes were lower while that of AA genotype was higher in patients (2.2%, 35.7%, 62.1%, respectively) than those in controls (8%, 42.0%, 50.0%, respectively). Meanwhile, the differences of A carriers with non-carriers and C carriers with non-carriers between the two groups were significant as well (*P* < 0.05). In addition, the distributions of C carriers and non-carriers of rs12795805 was found to be different between patients and controls (*P* < 0.05) in which the proportion of C carriers were significantly higher in patients, but this difference was no longer significant after Bonferroni correction. There was no significant difference among genotype frequencies and distributions of allele carriers vs non-carriers in rs10789970, rs4938056 and rs12421126 between the two groups. The comparison of the genotype frequencies and distribution of allele carriers vs non-carriers are shown in Tables 3 and 4.

**Table 3:** Comparison of genotype frequencies of *HTR3B gene* polymorphism loci between case and control groups

SNP(A1/A2)	Group	genotype(%)			χ^2^	*P*
		A1A1	A1A2	A2A2		
rs10789970 (C/T)	case (227)	39(17.2)	113(49.8)	75(33.0)	0.766	0.682
	control (200)	32(16.0)	108(54.0)	60(30.0)		
rs4938056 (C/T)	case (229)	43(18.8)	113(49.3)	73(31.9)	1.123	0.570
	control (202)	34(16.8)	110(54.5)	58(28.7)		
rs12421126 (C/T)	case (201)	8(4.0)	67(33.3)	126(62.7)	2.278	0.320
	control (176)	4(2.3)	70(39.8)	102(58.0)		
**rs1176744** **(A/C)**	case (227)	141(62.1)	81(35.7)	5(2.2)	**11.129**	0.004^*^
	control (200)	100(50.0)	84(42.0)	16(8.0)		
rs12795805 (C/T)	case (227)	6(2.6)	73(32.2)	148(65.2)	4.852	0.088
	control (200)	4(2.0)	46(23.0)	150(75.0)		

Bolded indicates statistically significant (*P* < 0.05)

^*^indicates the difference was still significant after Bonferroni correction

**Table 4:** Comparison on frequencies of allele carriers and non-carriers between groups

SNP(A1/A2)	Group	genotype		χ^2^_1_	*P*_*1*_	genotype		χ^2^_2_	*P*_*2*_
		A1A1 + A1A2	A2A2			A2A2 + A1A2	A1A1		
rs10789970	case (227)	152	75	0.454	0.500	188	39	0.107	0.744
(C/T)	control (200)	140	60			168	32		
rs4938056	case (229)	156	73	0.508	0.476	186	43	0.277	0.599
(C/T)	control (202)	144	58			168	34		
rs12421126	case (201)	75	126	0.879	0.348	193	8	0.888	0.346
(C/T)	control (176)	74	102			172	4		
rs1176744	case (227)	222	5	7.642	**0.006**	86	141	6.347	**0.012**
(A/C)	control (200)	184	16			100	100		
rs12795805	case (227)	79	148	4.845	**0.028**	221	6	0.192	0.661
(C/T)	control (200)	50	150			196	4		

Bolded indicates statistically significant (*P* < 0.05). χ^2^_1_: χ^2^[(A1A1 + A1A2)/A2A2]; χ^2^_2_: χ^2^[(A2A2 + A1A2)/A1A1]; *P*_*1*_: *P*[(A1A1 + A1A2)/A2A2]; *P*_*2*_: *P*[(A2A2 + A1A2)/A1A1]

#### Allelic distributions of *HTR3B* gene in patients and controls

There were statistical differences in the allelic distributions of rs1176744 and rs12795805 in *HTR3B* gene (χ^2^ = 9.288, *Ρ* = 0.002; χ^2^ = 4.256, *Ρ* = 0.039, respectively) between the two groups, and the proportions of A allele of rs1176744 and C allele of rs12795805 were significantly higher in patients. These differences were positively correlated with depressive disorder (OR = 1.126, 95% CI: 1.042 to 1.217; OR = 1.387, 95% CI: 1.014 to 1.897, respectively). However, *P* value of rs12795805 did not withstand Bonferroni correction for multiple testing. No association was found between depression and the other three SNPs tested. The comparison of allelic distributions of five polymorphic loci in *HTR3B* gene is presented in Table 5.

**Table 5:** Comparison of allele frequencies of five SNPs in *HTR3B* gene between the two groups

SNP	Al	Allele frequency		A2	χ^2^	*P*	OR	95%CI	
		Case group	Control group						
rs10789970	C	0.421	0.430	T	0.075	0.784	0.978	0.837	1.144
rs4938056	C	0.434	0.441	T	0.032	0.857	0.986	0.847	1.148
rs12421126	C	0.206	0.222	T	0.256	0.613	0.932	0.708	1.225
**rs1176744**	A	0.800	0.710	C	9.288	0.002^*^	**1.126**	1.042	1.217
**rs12795805**	C	0.187	0.135	T	4.256	**0.039**	1.387	1.014	1.897

Bolded indicates statistically significant (*P* < 0.05)

^*^indicates the difference was still significant after Bonferroni correction; OR value: hazard ratio; 95% CI: lower and upper limits of the confidence interval

#### Linkage disequilibrium and haplotype analysis

**Fig. 1:**
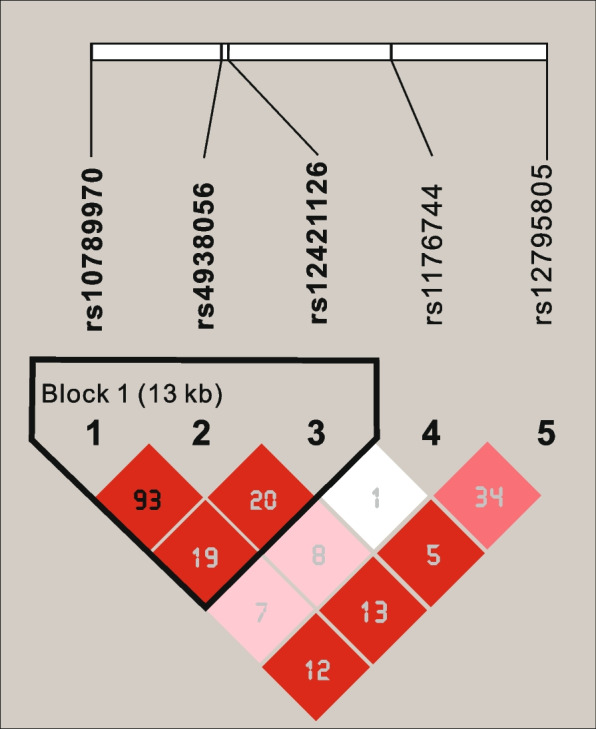
Linkage disequilibrium analysis of the selected SNPs. (Note: The numbers in the LD block are the values of R2 which are shown in the percentage chart. For example, 34 represents R2 = 0.34)

Linkage disequilibrium (LD) was determined between the five SNPs of *HTR3B* gene in both patients and controls. LD blocks of *HTR3B* gene SNPs and values of the correlation coefficient ${R}^{2}$ for both groups are presented in Fig. 1. Significant *P* values are shown in red on the haplotype block model created by the Haploview tool. One haplotype block was noteworthy, according to haplotype analysis employing the *HTR3B* gene loci that were under investigation. Between the three loci of this haplotype block, rs4938056, rs10789970, and rs12421126 were in linkage disequilibrium (Fig. 1).

### Relationship between *HTR3B* gene and cognitive deficits in patients

#### Comparison of cognitive functions between patients and controls

In this study, we first investigated the difference of cognitive functions examined between patients and controls. A total of five tests including Maze Test, Symbolic Coding Test, Spatial Span Inverse Order Test, Linking Test and Emotion Management Test were used to assess the executive functioning of all the participants. Significant differences were obtained in the scores of LT, SSIOT and EMT (*P* < 0.05), which indicated worse executive functions of depressed patients (Table 6). There was no difference in the scores of SCT and MT between groups (*P* > 0.05).

**Table 6:** Comparison of cognitive functions between case and control groups

Cognitive test items	Case group(229)	Control group(202)	t	*P*
	Mean ± standard deviation	Mean ± standard deviation		
LT	47.131 ± 22.479	43.820 ± 6.953	2.122	**0.035**
SCT	33.825 ± 11.528	35.391 ± 13.642	-1.291	0.197
MT	7.424 ± 1.611	7.510 ± 1.774	-0.529	0.597
SSIOT	5.148 ± 1.919	6.738 ± 1.800	-8.871	**0.000**
EMT	7.924 ± 1.654	8.477 ± 2.115	-2.990	**0.003**

Bolded indicates statistically significant (*P* < 0.05)

*LT* Linking Test, *SCT* Symbolic Coding Test, *MT* Maze Test, *SSIOT* Spatial Span Inverse Order Test, *EMT* Emotion Management Test

#### Potential confounders of cognitive deficits in patients

The potential confounders (age, education, gender, and total HAMD scores) of the impaired executive functions in patients were then investigated. The results showed that the scores of SSIOT were significantly associated with age (Table 7).

**Table 7:** Association between general information and cognitive deficits in patients

Dependent variable	Age		Years of education		Gender		Total HAMD score	
	r	*P*	F	*P*	F	*P*	r	*P*
LT	0.085	0.201	1.021	0.412	0.670	0.414	1.101	0.128
SSIOT	-0.167	**0.011**	1.086	0.372	0.038	0.846	0.002	0.982
EMT	-0.127	0.056	0.999	0.427	0.448	0.504	0.051	0.443

Bolded indicates statistically significant (*P* < 0.05)

*LT* Linking Test, *SSIOT* Spatial Span Inverse Order Test, *EMT* Emotion Management Test, r: Pearson correlation coefficient; F: the test statistic for the one-way ANOVA

#### Association of the polymorphism of *HTR3B* gene and cognitive deficits

According to the above results, covariance analysis in which age was regarded as covariate was then performed to find out whether different genotypes and allele carriers at different loci of *HTR3B* gene were related to cognitive deficits of depressive patients. The results revealed significant difference (*P* < 0.05) among three different genotypes and between different allele carriers of rs1176744 and rs12795805 in SSIOT. Patients with AA genotypes of rs1176744 had significantly lower scores, suggesting worse executive function, than those with AC and CC genotypes (*P* < 0.05).The A carriers had lower scores and C carriers had higher scores than non-carriers respectively, indicating A allele is a risk factor while C allele is a protective factor for executive function in patients. In addition, C carriers of rs12795805 had significant higher scores than non-carriers, demonstrating C allele is a protective factor (Table 8).

**Table 8:** Comparison of the correlations between genotype and allele carriers and cognitive function at different loci

SNP(A1/A2)	rs1176744(A/C)			rs10789970 (C/T)			rs4938056(C/T)		
	LT	SSIOT	EMT	LT	SSIOT	EMT	LT	SSIOT	EMT
A1A1	46.922±23.457	4.577±0.148	7.768±1.753	46.744±21.631	4.937±0.306	8.059±1.520	46.256±21.139	4.820±0.290	8.031±1.459
A1A2	47.321±21.661	6.004±0.196	8.184±1.516	48.434±25.133	5.037±0.179	7.886±1.646	48.726±25.331	5.144±0.178	7.906±1.661
A2A2	48.800±6.140	7.438±0.784	8.244±0.147	45.293±18.660	5.428±0.220	7.922±1.769	45.178±18.248	5.346±0.222	7.888±1.770
A1A1+A1A2	47.068±22.770	5.098±0.126	7.920±1.678	48.000±24.224	5.004±1.886	7.930±1.611	48.045±24.207	5.048±1.907	7.941±1.604
A2A2+A1A2	47.407±21.059	6.126±1.930	8.187±1.471	47.181±22.770	5.207±1.937	7.900±1.691	46.652±22.939	5.236±1.980	7.835±1.676
F	0.022	21.134	1.709	0.442	1.238	0.157	0.590	1.038	0.110
t_1_	-0.170	-5.478	-0.430	0.851	-1.627	0.037	0.899	-1.148	0.221
t_2_	0.157	6.262	1.851	0.110	0.987	-0.543	-0.710	1.540	-1.815
*P*	0.978	**0.000**	0.183	0.644	0.292	0.855	0.555	0.356	0.896
*P*_*1*_	0.899	**0.000**	0.086	0.687	0.778	0.574	0.541	0.342	0.691
*P*_*2*_	0.855	**0.000**	0.523	0.745	0.195	0.673	0.804	0.151	0.653
*P*_*3*_	0.887	0.078	0.915	0.352	0.170	0.890	0.295	0.479	0.919
*P*_*4*_	0.865	**0.003**	0.667	0.396	0.105	0.971	0.370	0.252	0.826
*P*_*5*_	0.875	**0.000**	0.065	0.912	0.325	0.588	0.478	0.125	0.071

SNP(A1/A2)	rs12421126(C/T)			rs12795805(C/T)		
	LT	SSIOT	EMT	LT	SSIOT	EMT
A1A1	41.750±14.210	4.124±0.648	8.734±1.888	49.167±18.115	6.320±0.764	8.005±0.518
A1A2	44.910±23.469	4.805±0.223	7.895±1.777	46.699±21.838	5.612±0.219	8.021±1.706
A2A2	48.984±23.368	5.221±0.163	7.961±1.631	47.223±23.142	4.873±0.154	7.878±1.673
A1A1+A1A2	44.516±23.368	4.972±2.093	7.918±1.744	46.886±21.486	5.682±1.732	8.020±1.644
A2A2+A1A2	47.230±22.767	5.174±1.929	7.900±1.656	47.050±22.671	5.117±1.923	7.925±1.681
F	0.921	2.157	0.884	0.039	5.006	0.188
t_1_	-1.486	-1.170	-0.050	-0.107	3.223	0.614
t_2_	0.482	0.892	-0.962	-0.227	-1.530	-0.116
*P*	0.400	0.118	0.415	0.962	**0.007**	0.873
*P*_*1*_	0.715	0.321	0.190	0.798	0.374	0.947
*P*_*2*_	0.392	0.102	0.211	0.837	0.065	0.812
*P*_*3*_	0.246	0.134	0.818	0.871	**0.006**	0.624
*P*_*4*_	0.139	0.243	0.960	0.915	**0.002**	0.549
*P*_*5*_	0.631	0.373	0.337	0.821	0.127	0.908

Bolded indicates statistically significant (*P* < 0.05); F: F(A1A1/A1A2/A2A2); t_1_: t[(A1A1 + A1A2)/A2A2]; t_2_: t[(A2A2 + A1A2)/A1A1]; *P*: *P*(A1A1/A1A2/A2A2); *P*_*1*_: *P*(A1A1/A1A2); *P*_*2*_*: P*(A1A1/ A2A2); *P*_*3*_*: P*(A1A2/A2A2); *P*_*4*_*: P*[(A1A1 + A1A2)/A2A2]; *P*_*5*_*: P*[(A1A1 + A1A2)/A1A1]; LT: Linking Test; SSIOT: Spatial Span Inverse Order Test; EMT: Emotion Management Test

## Discussion

This study had two goals. First, we investigated the relationship between the *HTR3B* gene polymorphisms and depressive disorder. We discovered that in the Chinese Han population, the polymorphism of rs1176744 in *HTR3B* gene may be associated with depressive disorder. Next, we investigated the connection between the polymorphisms of *HTR3B* gene and the executive dysfunction of patients. The findings demonstrated that rs1176744 and rs12795805 polymorphisms were associated with the scores of SSIOT. The A allele of rs1176744 may increase the risk of cognitive dysfunction and C alleles of rs1176744 and rs12795805 may be the protective factors in those with depressive disorder.

In this study, we found that there were more participants with AA genotype and A carriers while less C carriers of rs1176744 in patients than those in controls. The result was consistent with previous studies. Yamada et al. found that the rs1176744 locus cause missense mutations (Tyr129Ser:386A > C) and that Tyr129 homozygote (AA genotype) were more common in female depressed patients than controls [22]. In a 2019 study of 222 depressed and 147 healthy Russians [23], the rs1176744 polymorphism of *HTR3B* gene was also found to be significantly related with depression. A previous study showed that the heterozygote for the rs1176744 locus of AC (Y129S) could widen the maximum response to 5-HT, decrease desensitization and deactivation kinetics, and increase mean channel open duration [30]. In the same year, Karen's research revealed that the average opening time of the cation channel increased sevenfold in the Y129S receptor when compared with the AA genotype, and the increased signal displayed by the Y129S receptor may have a preventive effect on the onset of depression [31]. As a result, it could be suggested that AA genotype may be the risk factor for the etiology of depression and C allele may be a protective factor.

Our study found evidence about the association between the *HTR3B* gene polymorphism and the executive dysfunction of patients. Studies have reported that the mRNA of the *HTR3B* gene is mainly expressed in the prefrontal cortex, brainstem amygdala and mediodorsal thalamus, all of which have been shown to be associated with cognitive function [32–37]. There were few previous studies on the association of *5-HT3B* gene and cognitive function. Kulkarni J, et al. found that individuals with the C allele of SNP rs1176744 had lower scores on the Pain Catastrophizing Scale (PCS), suggesting that C allele may act as a preventive measure against pain catastrophizing, a coping strategy characterized by an excess of negative thoughts and feelings [38]. It has been well studied that there were parallel pathways between pain and depression [39], and our findings suggested that among depressed patients, the A carriers had worse performance and C carriers had better performance of executive function than non-carriers respectively, indicating that A allele may increase the risk of executive dysfunction while C allele showed a protective effect.

Another novel finding of this study was the results regarding the polymorphism of rs12795805. Association analyses demonstrated moderate correlation between C allele and depression (OR = 1.387) although the significance was no longer present after Bonferroni correction. It is important to note that the small effect size for this association could indicate a link between rs12795805 and depression, but that the effect size was too small to detect a significant difference with the current sample. This smaller effect size may have occurred due to an insufficient sample size. Meanwhile, C allele was found to have a protective effect on the executive dysfunction of depression. We have not found any previous study reporting the relationship between this locus and depression. Two recent studies on bipolar disorder and heroin addiction respectively observed no significant association of rs12795805 polymorphism with these two mental health problems [40, 41].

The relationship between 5-HT3 receptors and cognitive function have been investigated in different studies [26–28, 42–44] which revealed that 5-HT3 receptor antagonist could improve cognitive functions including attention, learning and memory. A recent study reviewing key data obtained from preclinical behavioral models and clinical trials of MDD concluded that vortioxetine (a 5-HT3 receptor antagonist) improved cognitive impairment in patients with MDD [45]. In a case–control research conducted in 2021, ondansetron, a serotonin (5-HT) 3 receptor antagonist, was found to increase MCCB scores (assessments included speed of processing, attention/alertness, working memory, verbal learning, visual learning, reasoning/problem-solving, and social cognition) in patients with schizophrenia [46].

Our findings are subject to several limitations. Firstly, the sample size was modest and the genetic power of this study was relatively low. Secondly, patients who chose not to seek treatment could not be involved in our findings, as our patients were identified from the outpatient and inpatient departments of the hospital. Thirdly, only the executive function was investigated, so the cognitive deficits of patients such as attention, learning and memory may not be fully explored. Lastly, a potential confound to findings of executive function is that depressed patients were allowed to be on antidepressant medications. We could not exclude the possibility that antidepressants may play a role in the current executive function findings and, if so, in which direction. For example, whilst some studies suggested that antidepressants may lead to improvement on executive function in MDD patients [47–49], this effect was not consistently observed, with a recent study concluding that the use of antidepressants, particularly SSRIs and trazodone, may instead increase the risk of cognitive impairment 5 years later among the oldest old women [50]. The current study adopted a real-world, pragmatic design, as requiring patients to wean off medications would likely have worsened their clinical condition and may have placed them at risk, including risk of suicide.

The etiology of depression is still unknown, and the pathogenesis of this disease could be caused by a variety of factors including genetics, gene expression, personality basis, social environment, etc. Therefore, further genetic and epigenetic studies based on larger sample sizes and studies to explore the interaction of genes and environments are needed.

## Conclusions

In our study, we found that the polymorphism of rs1176744 in *HTR3B* gene may be associated with depression, where individuals carrying A allele may be more likely to develop depressive disorder. The polymorphisms of rs1176744 and rs12795805 were associated with executive function, in which A allele of rs1176744 may increase the risk of cognitive dysfunction while C alleles of rs1176744 and rs12795805 may be the protective factors in patients with depression.

## Data Availability

Researchers interested in the study may contact corresponding author to obtain relevant data via email: qiaovincent@163.com.

## References

[1] Collins PY, Patel V, Joestl SS, March D, Insel TR, Daar AS (2011). Grand challenges in global mental health. Nature.

[2] Kindleberger CP. The world in depression, 1929–1939: Univ of California Press; 1986.

[3] Organization WH. Depression and other common mental disorders: global health estimates. World Health Organization; 2017.

[4] Woo YS, Rosenblat JD, Kakar R, Bahk WM, McIntyre RS (2016). Cognitive deficits as a mediator of poor occupational function in remitted major depressive disorder patients. Clin Psychopharmacol Neurosci.

[5] Association AP. DSM 5 diagnostic and statistical manual of mental disorders. DSM 5 Diagnostic and statistical manual of mental disorders2013. p. 947 p.- p.

[6] Chamberlain SR, Sahakian BJ (2004). Cognition in mania and depression: psychological models and clinical implications. Curr Psychiatry Rep.

[7] Semkovska M, Quinlivan L, O'Grady T, Johnson R, Collins A, O'Connor J (2019). Cognitive function following a major depressive episode: a systematic review and meta-analysis. The Lancet Psychiatry.

[8] McIntyre RS, Cha DS, Soczynska JK, Woldeyohannes HO, Gallaugher LA, Kudlow P (2013). Cognitive deficits and functional outcomes in major depressive disorder: determinants, substrates, and treatment interventions. Depress Anxiety.

[9] Knight MJ, Air T, Baune BT (2018). The role of cognitive impairment in psychosocial functioning in remitted depression. J Affect Disord.

[10] Withall A, Harris L, Cumming S (2009). The relationship between cognitive function and clinical and functional outcomes in major depressive disorder. Psychol Med.

[11] Sullivan PF, Neale MC, Kendler KS (2000). Genetic epidemiology of major depression: review and meta-analysis. Am J Psychiatry.

[12] Nishino J, Ochi H, Kochi Y, Tsunoda T, Matsui S (2018). Sample size for successful genome-wide association study of major depressive disorder. Front Genet.

[13] Jeon SW, Kim YK (2016). Neuroinflammation and cytokine abnormality in major depression: cause or consequence in that illness?. World J Psychiatry.

[14] Mekli K. Investigation of serotonergic receptors and transporter genes in vulnerability to depression, anxiety and neuroticism: A human population study. 2010.

[15] Barnes NM, Neumaier JF (2011). Neuronal 5-HT receptors and SERT. Tocris Biosci Sci Rev Ser.

[16] Faerber L, Drechsler S, Ladenburger S, Gschaidmeier H, Fischer W (2007). The neuronal 5-HT3 receptor network after 20 years of research–evolving concepts in management of pain and inflammation. Eur J Pharmacol.

[17] Villas-Boas GR, Lavorato SN, Paes MM, de Carvalho PMG, Rescia VC, Cunha MS, et al. Modulation of the Serotonergic Receptosome in the Treatment of Anxiety and Depression: A Narrative Review of the Experimental Evidence. Pharmaceuticals (Basel). 2021;14(2). 10.3390/ph14020148 PMC7918669 33673205

[18] Pytka K, Gluch-Lutwin M, Kotanska M, Waszkielewicz A, Kij A, Walczak M (2018). Single Administration of HBK-15-a Triple 5-HT1A, 5-HT7, and 5-HT3 receptor antagonist-reverses depressive-like behaviors in mouse model of depression induced by corticosterone. Mol Neurobiol.

[19] Mitchell EA, Pratt JA (1991). Neuroanatomical structures involved in the action of the 5-HT3 antagonist ondansetron: a 2-deoxyglucose autoradiographic study in the rat. Brain Res.

[20] Niesler B, Kapeller J, Hammer C, Rappold G. Serotonin type 3 receptor genes: Htr3a, b, c, d, e. 2008. 10.2217/14622416.9.5.501 18466097

[21] Dubin AE, Huvar R, D'Andrea MR, Pyati J, Zhu JY, Joy KC (1999). The pharmacological and functional characteristics of the serotonin 5-HT(3A) receptor are specifically modified by a 5-HT(3B) receptor subunit. J Biol Chem.

[22] Yamada K, Hattori E, Iwayama Y, Ohnishi T, Ohba H, Toyota T (2006). Distinguishable haplotype blocks in the HTR3A and HTR3B region in the Japanese reveal evidence of association of HTR3B with female major depression. Biol Psychiatry.

[23] Vyalova NM, Simutkin GG (2019). Association of polymorphic variants of serotonin receptor genes, serotonin synthesis and metabolism enzymes genes with depressive disorder and clinical remission. VM Bekhterev Review of Psychiatry and Medical Psychology.

[24] Frank B, Niesler B, Nothen MM, Neidt H, Propping P, Bondy B (2004). Investigation of the human serotonin receptor gene HTR3B in bipolar affective and schizophrenic patients. Am J Med Genet B Neuropsychiatr Genet.

[25] Hammer C, Cichon S, Muhleisen TW, Haenisch B, Degenhardt F, Mattheisen M (2012). Replication of functional serotonin receptor type 3A and B variants in bipolar affective disorder: a European multicenter study. Transl Psychiatry.

[26] Carey G, Costall B, Domeney A, Gerrard P, Jones D, Naylor R (1992). Ondansetron and arecoline prevent scopolamine-induced cognitive deficits in the marmoset. Pharmacol Biochem Behav.

[27] Boast C, Bartolomeo AC, Morris H, Moyer JA (1999). 5HT antagonists attenuate MK801-impaired radial arm maze performance in rats. Neurobiol Learn Mem.

[28] Pitsikas N, Borsini F (1996). Itasetron (DAU 6215) prevents age-related memory deficits in the rat in a multiple choice avoidance task. Eur J Pharmacol.

[29] Liang S, Yu W, Ma X, Luo S, Zhang J, Sun X (2020). Psychometric properties of the MATRICS Consensus Cognitive Battery (MCCB) in Chinese patients with major depressive disorder. J Affect Disord.

[30] Walstab J, Hammer C, Bonisch H, Rappold G, Niesler B (2008). Naturally occurring variants in the HTR3B gene significantly alter properties of human heteromeric 5-hydroxytryptamine-3A/B receptors. Pharmacogenet Genomics.

[31] Krzywkowski K, Davies PA, Feinberg-Zadek PL, Brauner-Osborne H, Jensen AA (2008). High-frequency HTR3B variant associated with major depression dramatically augments the signaling of the human 5-HT3AB receptor. Proc Natl Acad Sci U S A.

[32] Sakurai T, Gamo NJ (2019). Cognitive functions associated with developing prefrontal cortex during adolescence and developmental neuropsychiatric disorders. Neurobiol Dis.

[33] Ouhaz Z, Fleming H, Mitchell AS (2018). Cognitive functions and neurodevelopmental disorders involving the prefrontal cortex and mediodorsal thalamus. Front Neurosci.

[34] Friedman NP, Robbins TW (2022). The role of prefrontal cortex in cognitive control and executive function. Neuropsychopharmacology.

[35] Tang S, Li H, Lu L, Wang Y, Zhang L, Hu X (2019). Anomalous functional connectivity of amygdala subregional networks in major depressive disorder. Depress Anxiety.

[36] Leiser SC, Li Y, Pehrson AL, Dale E, Smagin G, Sanchez C (2015). Serotonergic regulation of prefrontal cortical circuitries involved in cognitive processing: a review of individual 5-HT receptor mechanisms and concerted effects of 5-HT receptors exemplified by the multimodal antidepressant vortioxetine. ACS Chem Neurosci.

[37] Sargin D, Jeoung HS, Goodfellow NM, Lambe EK (2019). Serotonin regulation of the prefrontal cortex: cognitive relevance and the impact of developmental perturbation. ACS Chem Neurosci.

[38] Horjales-Araujo E, Demontis D, Lund EK, Finnerup NB, Borglum AD, Jensen TS (2013). Polymorphism in serotonin receptor 3B is associated with pain catastrophizing. PLoS ONE.

[39] Delgado PL (2004). Common pathways of depression and pain. J Clin Psychiatry.

[40] Jian J, Li C, Xu J, Qiao D, Mi G, Chen X, et al. Associations of serotonin receptor gene HTR3A, HTR3B, and HTR3A haplotypes with bipolar disorder in Chinese patients. Genet Mol Res. 2016;15. 10.4238/gmr.15038671 27706728

[41] Yin F, Ji Y, Zhang J, Guo H, Huang X, Lai J (2016). Polymorphisms in the 5-hydroxytryptamine receptor 3B gene are associated with heroin dependence in the Chinese Han population. Neurosci Lett.

[42] Lennertz L, Wagner M, Frommann I, Schulze-Rauschenbach S, Schuhmacher A, Kuhn KU (2010). A coding variant of the novel serotonin receptor subunit 5-HT3E influences sustained attention in schizophrenia patients. Eur Neuropsychopharmacol.

[43] Terry AV, Buccafusco JJ, Wilson C (2008). Cognitive dysfunction in neuropsychiatric disorders: selected serotonin receptor subtypes as therapeutic targets. Behav Brain Res.

[44] Arnsten AF, Lin CH, Van Dyck CH, Stanhope KJ (1997). The effects of 5-HT3 receptor antagonists on cognitive performance in aged monkeys. Neurobiol Aging.

[45] Bennabi D, Haffen E, Van Waes V (2019). Vortioxetine for cognitive enhancement in major depression: from animal models to clinical research. Front Psychiatry.

[46] Kulkarni J, Thomas N, Hudaib A, Gavrilidis E, Gurvich C (2021). Ondansetron–a promising adjunctive treatment for persistent schizophrenia. Biol Psychiat.

[47] Pimontel MA, Rindskopf D, Rutherford BR, Brown PJ, Roose SP, Sneed JR (2016). A meta-analysis of executive dysfunction and antidepressant treatment response in late-life depression. Am J Geriatr Psychiatry.

[48] Basso L, Bönke L, Aust S, Gärtner M, Heuser-Collier I, Otte C (2020). Antidepressant and neurocognitive effects of serial ketamine administration versus ECT in depressed patients. J Psychiatr Res.

[49] Gudayol-Ferré E, Duarte-Rosas P, Peró-Cebollero M, Guàrdia-Olmos J (2021). The effect of second-generation antidepressant treatment on the executive functions of patients with major depressive disorder: a meta-analysis study with structural equation models. Psychiatry Res.

[50] Leng Y, Diem SJ, Stone KL, Yaffe K (2018). Antidepressant use and cognitive outcomes in very old women. The Journals of Gerontology: Series A.

